# Prior Image-Guided Adaptive-Weighted Relative Total Variation for Sparse-View Computed Laminography of Plate-like Objects

**DOI:** 10.3390/s26144519

**Published:** 2026-07-16

**Authors:** Jing Lu, Shu Li, Hangqi Wu, Yongxing Pei

**Affiliations:** 1 Department of Automation, Taiyuan Institute of Technology, Taiyuan 030008, China; 2 State Key Laboratory of Extreme Environment Optoelectronic Dynamic Testing Technology and Instrument, North University of China, Taiyuan 030051, China; nuc_ls@163.com (S.L.); hangqi_wu@163.com (H.W.); 3 Taiyuan Satellite Launch Center, Taiyuan 030017, China; pyx1019411736@163.com

**Keywords:** X-ray, computed laminography, prior image, adaptive weighting, relative total variation

## Abstract

X-ray computed laminography (CL) is a promising nondestructive testing technique for plate-type objects and is of great importance in 3D imaging. Nevertheless, its scanning geometry results in a lack of projection data along in-plane directions, causing severe inter-slice aliasing and cone-beam artifacts, especially under sparse-view sampling. To address this challenge, a prior image-guided adaptive-weighted relative total variation (PiAwRTV) algorithm is proposed for sparse-view CL. Based on relative total variation (RTV), PiAwRTV leverages structural information from a high-quality prior image to guide image reconstruction and introduces weights that vary with local image gradients. The reconstruction model incorporates 2D PiAwRTV in the horizontal direction to perform edge-preserving smoothing and 1D PiAwRTV in the vertical direction to suppress inter-slice blurring. An alternating minimization strategy is employed to decompose this optimization problem into three subproblems for iterative solution. Experimental results demonstrate that the proposed algorithm reconstructs key structural features while reducing cone-beam artifacts, significantly improving the imaging quality of sparse-view CL.

## 1. Introduction

**Figure 1:**
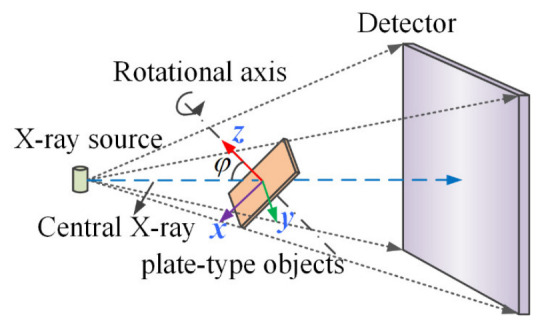
Schematic diagram of the geometry of the CL system.

In modern high-end manufacturing, internal structure imaging of integrated circuits, multi-layer printed circuit boards (PCBs), and large composite material components [1,2,3] has become a critical step in ensuring product quality and reliability. However, conventional X-ray computed tomography (CT) is limited by its requirement for 360° rotation, making it unsuitable for inspecting objects with a large aspect ratio. There are two main reasons. First, achieving high spatial resolution requires the object to be positioned near the X-ray source, which may result in a collision between the planar sample and the source. Second, planar objects typically exhibit high X-ray attenuation along in-plane directions, which hinders X-ray penetration and results in a low signal-to-noise ratio. To overcome these drawbacks, computed laminography (CL) has been developed as an alternative. The geometric configurations of CL systems are generally categorized as planar [4,5], swing [6], and rotational [7,8]. Rotational CL provides richer projection data, offers better adaptability to fixed source–detector configurations, and ensures consistent spatial resolution in both the *x* and *y* directions. The geometry of a rotational CL system is illustrated in Figure 1. The X-ray source and detector remain stationary, while the plate-like object rotates about an axis perpendicular to its surface. The object’s rotation axis is tilted at an angle φ relative to the central X-ray beam, which not only avoids collision but also confines the X-ray propagation path length to the thickness of the object, thereby greatly reducing the required X-ray energy. While CL enables imaging of planar objects, its geometric configuration leads to incomplete projection data. When conventional reconstruction methods, such as Feldkamp–Davis–Kress (FDK) [9], algebraic reconstruction technique (ART) [10], or simultaneous ART (SART) [11], are applied to CL data, they tend to introduce inter-slice aliasing and cone-beam artifacts in the reconstructed images, manifesting as edge blurring and structural degradation along the vertical direction. Sparse-view sampling is often employed to shorten scanning time, yet the reduction in projection data further exacerbates reconstruction artifacts. Therefore, achieving high-quality sparse-view CL reconstruction has become a challenging research topic in nondestructive testing.

Iterative algorithms that incorporate additional prior knowledge have demonstrated advantages in image reconstruction, such as total variation (TV) [12,13], low-rank [14,15], wavelet tight frame [16,17], dictionary learning [18,19], and curvelet and shearlet transform [20,21]. By exploiting image sparsity in a transform domain, these methods constrain the solution space, enabling the reconstruction of high-quality images from incomplete projection data. As a representative gradient-based regularization method, TV has been widely adopted due to its concise formulation and computational efficiency. However, standard TV assumes images to be piecewise constant, which tends to over-smooth edges and lose fine details. Moreover, it fails to suppress the directional artifacts of CL due to its isotropic nature. Subsequent studies have improved TV from two perspectives. In terms of adaptive weighting, strategies such as adaptive-weighted TV (AwTV) [22], relative TV (RTV) [23], and adaptive-weighted dynamic-adjusted RTV (AwDaRTV) [24] have been developed. Adaptive weights based on local image intensity gradients are introduced into these methods to preserve edges and fine details. In terms of directional weighting, methods such as anisotropic TV (ATV) [25], anisotropic alternating regularization (AAR) [26], and directional TV (DTV) [27] have been proposed. By encoding scanning geometry as a directional prior and imposing differential constraints on gradients in different directions, inter-slice blurring can be effectively alleviated. The fundamental difficulty of sparse-view CL reconstruction lies in the severe undersampling of projection data, which makes the solution space highly non-unique. Although the above methods have improved upon standard TV, they use only gradient statistics derived from intermediate images during iteration, making it difficult to distinguish real edges from spurious structures caused by inter-slice aliasing. Utilizing a high-quality prior image as an additional structural constraint can improve reconstruction quality under sparse-view sampling, motivating various prior image-guided methods. Prior image constrained compressed sensing (PICCS) [28] adopts the weighted sum of the target image’s TV and that of its difference from the prior image as regularization terms for sparse-view CT reconstruction. Prior image constrained total generalized variation (PICTGV) [29] replaces TV in PICCS with TGV, suppressing staircase artifacts while preserving fine details. Prior contour-based total variation (PCTV) [30] derives adaptive weights from edge contours extracted from the prior image, guiding TV-regularized reconstruction to enhance edge information.

Recently, deep learning has shown great potential in sparse-view CT reconstruction, with methods broadly falling into three categories: projection-domain completion, image-domain post-processing, and multi-domain joint processing. In the context of CL, several deep learning-based methods have been developed for image-domain post-processing tasks. These include artifact reduction using convolutional neural networks (CNNs) and U-Net architectures [31], motion artifact correction through multi-angle fusion networks [32], super-resolution reconstruction for laminographic images [33], and defect detection or segmentation using networks such as Swin–Condinst, SAM, and self-training segmentation frameworks [34,35,36]. However, despite these advances, deep learning research that addresses the CL reconstruction problem itself remains relatively limited, and most existing methods are confined to image-domain post-processing. This is primarily because the tilted detector geometry in CL is difficult to encode directly into the projection data, making projection-domain approaches less intuitive. Furthermore, deep learning-based methods typically require large-scale training datasets with ground truth, which are difficult and costly to acquire in CL imaging. Therefore, this paper adopts an iterative reconstruction method that ensures structural fidelity without requiring training data.

Inspired by the above research results and targeting the requirements of sparse CL reconstruction, this paper proposes a prior image-guided adaptive-weighted relative total variation (PiAwRTV) model. The main contributions of this work are threefold:

1. Prior-guided structural anchoring. Unlike existing RTV-based methods (e.g., AwDaRTV [24]) that rely solely on gradient statistics from the intermediate image, our PiAwRTV model introduces a high-quality prior image to precompute the windowed inherent variation (WIV). This provides a stable structural reference that helps distinguish true edges from inter-slice aliasing, which is beneficial under sparse-view sampling where intermediate iterations are often corrupted.
2. Adaptive anisotropic regularization. While PCTV [30] incorporates prior information using a contour-based TV model that tends to smooth fine details, our method leverages the structure–texture separation capability of RTV and further introduces adaptive weights derived from local image gradients to dynamically adjust the regularization strength. This strategy assigns weaker penalties to strong edges and stronger penalties to flat regions, helping to preserve fine structures that might otherwise be lost.
3. Decoupled 2D/1D regularization. Unlike our previous AAR method [26] that applies 1D regularization to all three directions and produces horizontal stripe artifacts, the proposed method applies 2D PiAwRTV to the horizontal direction and 1D PiAwRTV to the vertical direction. This decoupled design preserves in-plane structural correlations while suppressing inter-slice aliasing, overcoming the limitation of AAR.

Experimental results show that, under sparse-view sampling, the proposed POCS-PiAwRTV algorithm effectively suppresses inter-slice aliasing artifacts and accurately reconstructs the structures of planar objects while preserving fine details.

The remainder of this paper is organized as follows. Section 2 gives a brief review of related works. Section 3 presents the PiAwRTV-based CL reconstruction model and derives the solution algorithm. Section 4 validates the proposed method using simulated and real data. Section 5 discusses the parameter selection strategy for the proposed algorithm. Section 6 concludes this paper.

## 2. Related Works

### 2.1. CL Reconstruction Model

CL reconstruction is an inverse problem, which can be discretized into the following linear system:

(1) Af=p,

where $f=\left(f_{n}\right)\in {\mathbb{R}}^{N}$ denotes the vectorized image to be reconstructed, which corresponds to the 3D volume data $f=\left(f_{i,j,k}\right)\in {\mathbb{R}}^{N_{1}\times N_{2}\times N_{3}}$ with the index mapping $n=\left(k-1\right)\times N_{1}\times N_{2}+\left(j-1\right)\times N_{1}+i$. $p=\left(p_{m}\right)\in {\mathbb{R}}^{M}$ is the projection vector, $A=\left(a_{ij}\right)\in {\mathbb{R}}^{M\times N}$ is the system matrix, and its element is defined as the intersection length of the *i*th ray through the *j*th voxel in the 3D cone beam [37].

**Figure 2:**
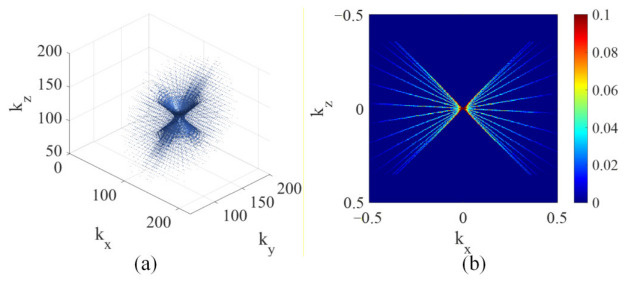
Fourier domain sampling distribution of the CL projection operator ***A***. (**a**) 3D isosurface. (**b**) Central slice at k_y_ = 0.

With M≪N, Equation (1) is underdetermined, and regularization is required to stabilize the solution. As shown in Figure 2, the Fourier domain sampling of the CL operator ***A*** has two missing cones, which directly causes inter-slice aliasing artifacts. Given the non-negativity of ***f***, the reconstruction model can be formulated as the following minimization problem:

(2) $$\underset{f\geq 0}{\arg\min}\left\{{\left\|Af-p\right\|}_{2}^{2}+\lambda \cdot R(f)\right\},$$

where ${\left\|Af-p\right\|}_{2}^{2}$ is the data fidelity term, R(f) is the regularization term, λ is the regularization parameter, and f≥0 denotes the non-negativity constraint.

### 2.2. RTV Regularization

RTV is defined as:

(3) $$\mathrm{RTV}(f)=\sum_{i}\left[\frac{D_{x}\left(f_{i}\right)}{L_{x}\left(f_{i}\right)+\varepsilon }+\frac{D_{y}\left(f_{i}\right)}{L_{y}\left(f_{i}\right)+\varepsilon }+\frac{D_{z}\left(f_{i}\right)}{L_{z}\left(f_{i}\right)+\varepsilon }\right],$$

where ε is a small positive constant to prevent division by zero. $D_{x}\left(f_{i}\right)$, $D_{y}\left(f_{i}\right)$, and $D_{z}\left(f_{i}\right)$ denote the windowed total variation (WTV) in the image at voxel *i* in the *x*, *y*, and *z* directions. $L_{x}\left(f_{i}\right)$, $L_{y}\left(f_{i}\right)$, and $L_{z}\left(f_{i}\right)$ denote the windowed inherent variation (WIV) in the image at voxel *i* in the *x*, *y*, and *z* directions. They are defined as:

(4) $$D_{x}\left(f_{i}\right)=\sum_{j\in W(i)}g_{i,j}\left|\partial_{x}f_{j}\right|,\;D_{y}\left(f_{i}\right)=\sum_{j\in W(i)}g_{i,j}\left|\partial_{y}f_{j}\right|,\;D_{z}\left(f_{i}\right)=\sum_{j\in W(i)}g_{i,j}\left|\partial_{z}f_{j}\right|,$$

(5) $$L_{x}\left(f_{i}\right)=\left|\sum_{j\in W(i)}g_{i,j}\cdot \partial_{x}f_{j}\right|,\;L_{y}\left(f_{i}\right)=\left|\sum_{j\in W(i)}g_{i,j}\cdot \partial_{y}f_{j}\right|,\;L_{z}\left(f_{i}\right)=\left|\sum_{j\in W(i)}g_{i,j}\cdot \partial_{z}f_{j}\right|,$$

where $W\left(i\right)$ denotes the local 3D window centered at voxel *i* and $g_{i,j}$ is a Gaussian weighting function given by:

(6) $$g_{i,j}\propto \exp\left(-\left({\left(x_{i}-x_{j}\right)}^{2}+{\left(y_{i}-y_{j}\right)}^{2}+{\left(z_{i}-z_{j}\right)}^{2}\right)/\left(2\sigma^{2}\right)\right),$$

where $\left(x_{i},y_{i},z_{i}\right)$ and $\left(x_{j},y_{j},z_{j}\right)$ denote the spatial coordinates of voxels *i* and *j* and σ is the standard deviation of the Gaussian function. The ratio of WTV to WIV is large at structural edges and small in textured regions, allowing RTV to distinguish structures from textures.

## 3. Proposed Method

### 3.1. PiAwRTV Regularization

The projection data acquired from sparse-view scanning are incomplete. The conventional RTV model relies solely on the image’s own gradient statistics, making it prone to misidentifying artifacts as structures and failing to capture true edges. Thus, the PiAwRTV model is proposed, which introduces a prior image $\tilde{f}$ to provide reliable structural priors and designs an adaptive weighting strategy to dynamically adjust the anisotropic penalty strength. PiAwRTV is defined as:

(7) $$\mathrm{PiAwRTV}(f)=\sum_{i}\left[\omega_{x}\left(f_{i}\right)\frac{D_{x}\left(f_{i}\right)}{L_{x}\left({\tilde{f}}_{i}\right)+\varepsilon }+\omega_{y}\left(f_{i}\right)\frac{D_{y}\left(f_{i}\right)}{L_{y}\left({\tilde{f}}_{i}\right)+\varepsilon }+\omega_{z}\left(f_{i}\right)\frac{D_{z}\left(f_{i}\right)}{L_{z}\left({\tilde{f}}_{i}\right)+\varepsilon }\right],$$

where $\omega_{x}\left(f_{i}\right)$, $\omega_{y}\left(f_{i}\right)$, and $\omega_{z}\left(f_{i}\right)$ are the adaptive weighting coefficients in the *x*, *y*, and *z* directions and are expressed as:

(8) $$\omega_{x}\left(f_{i}\right)=\exp\left(-\partial_{x}^{2}f_{i}/\delta^{2}\right),\;\omega_{y}\left(f_{i}\right)=\exp\left(-\partial_{y}^{2}f_{i}/\delta^{2}\right),\;\omega_{z}\left(f_{i}\right)=\exp\left(-\partial_{z}^{2}f_{i}/\delta^{2}\right),$$

where δ is a scale factor that controls the sensitivity of edge preservation. To incorporate prior structural information, the modified WIV in the denominator is defined as:

(9) $$L_{x}\left({\tilde{f}}_{i}\right)=\left|\sum_{j\in W(i)}h_{i,j}\cdot \partial_{x}{\tilde{f}}_{j}\right|,\;L_{y}\left({\tilde{f}}_{i}\right)=\left|\sum_{j\in W(i)}h_{i,j}\cdot \partial_{y}{\tilde{f}}_{j}\right|,\;L_{z}\left({\tilde{f}}_{i}\right)=\left|\sum_{j\in W(i)}h_{i,j}\cdot \partial_{z}{\tilde{f}}_{j}\right|.$$

The main advantages of PiAwRTV are twofold. First, the WIV values precomputed from the prior image $\tilde{f}$ with relatively accurate structures serve as pixel-wise weights to penalize image gradients, guiding structure recovery in sparse-view CL reconstruction. Second, the adaptive coefficients $\omega_{x}\left(f_{i}\right)$, $\omega_{y}\left(f_{i}\right)$, and $\omega_{z}\left(f_{i}\right)$ constructed via an exponential function dynamically adjust the penalty strength according to the intensity gradients in different directions. This strategy assigns stronger weights to small intensity variations in flat regions and weaker weights to large intensity variations in structural regions, thereby suppressing artifacts while preserving details with high fidelity.

### 3.2. PiAwRTV-Based Model

In our previous work, we proposed the AAR model [26], which introduces 1D regularization terms along three orthogonal directions and optimizes them alternately to enforce gradient sparsity in different directions, preserving in-plane edges and recovering inter-slice edges. However, the 1D regularization along the *x* and *y* directions disrupts the correlations among rows and among columns of the image, leading to horizontal stripe artifacts. Therefore, we introduce 2D PiAwRTV in the horizontal direction and 1D PiAwRTV in the vertical direction into the objective function as follows:

(10) $$\underset{f\geq 0}{\arg\min}\left\{{\left\|Af-p\right\|}_{2}^{2}+\lambda_{1}\cdot {\mathrm{PiAwRTV}}_{xy}(f)+\lambda_{2}\cdot {\mathrm{PiAwRTV}}_{z}(f)\right\},$$

where $\lambda_{1}$ and $\lambda_{2}$ are the weights that balance the two regularization terms. ${\mathrm{PiAwRTV}}_{xy}(f)$ is the 2D PiAwRTV in the horizontal direction, and ${\mathrm{PiAwRTV}}_{z}(f)$ is the 1D PiAwRTV in the vertical direction. They are expressed as:

(11) $${\mathrm{PiAwRTV}}_{xy}(f)=\sum_{i=1}\left[\omega_{x}\left(f_{i}\right)\frac{D_{x}\left(f_{i}\right)}{L_{x}\left({\tilde{f}}_{i}\right)+\varepsilon }+\omega_{y}\left(f_{i}\right)\frac{D_{y}\left(f_{i}\right)}{L_{y}\left({\tilde{f}}_{i}\right)+\varepsilon }\right],$$

(12) $${\mathrm{PiAwRTV}}_{z}(f)=\sum_{i=1}\left[\omega_{z}\left(f_{i}\right)\frac{D_{z}\left(f_{i}\right)}{L_{z}\left(f_{i}\right)+\varepsilon }\right].$$

### 3.3. POCS-PiAwRTV Algorithm

To solve model (10), an alternating minimization strategy is employed to decompose the original problem into three subproblems for iterative solution. Let $f^{\left(k\right)}$ denote the result at the *k*-th iteration. Then, the result at the (*k* + 1)-th iteration $f^{\left(k+1\right)}$ can be obtained by solving the following subproblems:

Subproblem 1:

(13) $$f^{\left(k+1/3\right)}=\underset{f\geq 0}{\arg\min}\left\{{\left\|f-f^{\left(k\right)}\right\|}_{2}^{2}+\lambda_{0}\cdot {\left\|Af-p\right\|}_{2}^{2}\right\},$$

Subproblem 2:

(14) $$f^{\left(k+2/3\right)}=\underset{f\geq 0}{\arg\min}\left\{{\left\|f-f^{\left(k+1/3\right)}\right\|}_{2}^{2}+\lambda_{1}\cdot {\mathrm{PiAwRTV}}_{xy}(f)\right\},$$

Subproblem 3:

(15) $$f^{\left(k+1\right)}=\underset{f\geq 0}{\arg\min}\left\{{\left\|f-f^{\left(k+2/3\right)}\right\|}_{2}^{2}+\lambda_{2}\cdot {\mathrm{PiAwRTV}}_{z}(f)\right\},$$

where $\lambda_{0}$ is a positive constant. Each subproblem is solved as follows.

(1) Solution of Subproblem 1

Subproblem 1 consists of a proximal term and a data fidelity term, where the latter imposes an additional constraint on the forward projection of $f^{\left(k\right)}$. First, with $f^{\left(k\right)}$ as the initial estimate, one iteration of SART is performed to obtain an intermediate result $f_{\mathrm{SART}}^{\left(k\right)}$. Then, the non-negativity constraint is imposed on $f_{\mathrm{SART}}^{\left(k\right)}$ to obtain $f^{\left(k+1/3\right)}$. This step is referred to as the projection onto convex sets (POCS) step, which can be expressed as:

(16) $$f_{\mathrm{SART}}^{\left(k\right)}=\mathrm{SART}\left(f^{\left(k\right)},p,\zeta \right),$$

(17) $$f^{\left(k+1/4\right)}=\mathrm{shrink}\left(f_{\mathrm{SART}}^{\left(k\right)},0\right),$$

where $\mathrm{SART}\left(f^{\left(k\right)},p,\zeta \right)$ represents one SART iteration with $f^{\left(k\right)}$ as the initial image, ***p*** is the projection data, and ζ is the relaxation parameter. $\mathrm{shrink}\left(\cdot ,0\right)$ is the hard thresholding function with threshold zero, defined as:

(18) $$\mathrm{shrink}\left(a,0\right)=\left\{\begin{array}{cc} a, & a\geq 0\\ 0, & a<0 \end{array}\right.,$$

For a vector ***a***, the function applies the thresholding operation element-wise.

(2) Solution of Subproblem 2

For Subproblem 2, ${\mathrm{PiAwRTV}}_{xy}(f)$ consists of components in the *x* and *y* directions. The *x*-direction component is decomposed into the product of a nonlinear term and a quadratic term as follows:

(19) $$\begin{array}{ll} \sum_{i}\omega_{x}\left(f_{i}\right)\frac{D_{x}\left(f_{i}\right)}{L_{x}\left({\tilde{f}}_{i}\right)+\varepsilon } & =\sum_{j}\sum_{i\in W(j)}\frac{\omega_{x}\left(f_{i}\right)g_{i,j}\left|\partial_{x}f_{j}\right|}{\left|\sum_{j\in \Omega (i)}h_{i,j}\cdot \partial_{x}{\tilde{f}}_{j}\right|+\varepsilon }\\ & \approx \sum_{j}\sum_{i\in W(j)}\frac{\omega_{x}\left(f_{i}\right)g_{i,j}}{L_{x}\left({\tilde{f}}_{i}\right)+\varepsilon }\frac{1}{\left|\partial_{x}f_{j}\right|+\xi }{\left(\partial_{x}f_{j}\right)}^{2}\\ & =\sum_{j}u_{x,j}w_{x,j}{\left(\partial_{x}f_{j}\right)}^{2} \end{array},$$

where ξ is a small positive constant to prevent numerical instability. $u_{x,j}$ and $w_{x,j}$ are defined as:

(20) $$u_{x,j}=\sum_{i\in W(j)}\frac{\omega_{x}\left(f_{i}\right)g_{i,j}}{L_{x}\left({\tilde{f}}_{i}\right)+\varepsilon },\;w_{x,j}=\frac{1}{\left|\partial_{x}f_{j}\right|+\xi },$$

The *y*-direction component can be expressed analogously as:

(21) $$\sum_{i}\omega_{y}\left(f_{i}\right)\frac{D_{y}\left(f_{i}\right)}{L_{y}\left({\tilde{f}}_{i}\right)+\varepsilon }=\sum_{j}u_{y,j}w_{y,j}{\left(\partial_{y}f_{j}\right)}^{2},$$

where $u_{y,j}$ and $w_{y,j}$ are defined as:

(22) $$u_{y,j}=\sum_{i\in W(j)}\frac{\omega_{y}\left(f_{i}\right)g_{i,j}}{L_{y}\left({\tilde{f}}_{i}\right)+\varepsilon },\;w_{y,j}=\frac{1}{\left|\partial_{y}f_{j}\right|+\xi },$$

Using matrix notation, Equation (14) can be written as:

(23) $$\underset{f\geq 0}{\arg\min}\left\{{\left(v_{f}-v_{f^{\left(k+1/3\right)}}\right)}^{T}\left(v_{f}-v_{f^{\left(k+3\right)}}\right)+\right.\left.\lambda_{1}\left(v_{f}^{T}C_{x}^{T}U_{x}W_{x}C_{x}v_{f}+v_{f}^{T}C_{y}^{T}U_{y}W_{y}C_{y}v_{f}\right)\right\},$$

where $v_{f}$ and $v_{f^{\left(k+1/3\right)}}$ are the vectorized forms of f and $f^{\left(k+1/3\right)}$. $C_{x}$ and $C_{y}$ are the Toeplitz matrices corresponding to the forward difference operators in the *x* and *y* directions. $U_{x}$, $U_{y}$, $W_{x}$, and $W_{y}$ are diagonal matrices whose diagonal entries are $U_{x}\left[j,j\right]=u_{x,j}$, $U_{y}\left[j,j\right]=u_{y,j}$, $W_{x}\left[j,j\right]=w_{x,j}$, and $W_{y}\left[j,j\right]=w_{y,j}$. The solution to Equation (23) can be obtained by iteratively solving the following linear equation:

(24) $$\left(I+\lambda_{1}\Phi_{xy}^{k,t}\right)v_{f^{\left(k+1/3\right)}}^{t+1}=v_{f^{\left(k+1/3\right)}},$$

where ***I*** is the identity matrix. $\Phi_{xy}^{k,t}=C_{x}^{T}U_{x}W_{x}C_{x}+C_{y}^{T}U_{y}W_{y}C_{y}$ is the weight matrix of the intermediate result $v_{f^{\left(k+1/3\right)}}^{t+1}$ obtained after *t* iterations of Equation (24) starting from $f^{\left(1+1/3\right)}$, with $t=0,1,\ldots ,N_{xy}-1$, where $N_{xy}$ is the number of iterations for Subproblem 2. The matrix $\left(I+\lambda_{1}\Phi_{xy}^{k,t}\right)$ is symmetric positive definite, which ensures numerical stability.

(3) Solution of Subproblem 3

Subproblem 3 is solved similarly to Subproblem 2, with ${\mathrm{PiAwRTV}}_{z}(f)$ expressed as:

(25) $$\sum_{i}\omega_{z}\left(f_{i}\right)\frac{D_{z}\left(f_{i}\right)}{L_{z}\left({\tilde{f}}_{i}\right)+\varepsilon }=\sum_{j}u_{z,j}w_{z,j}{\left(\partial_{z}f_{j}\right)}^{2},$$

where $u_{z,j}$ and $w_{z,j}$ are defined as:

(26) $$u_{z,j}=\sum_{i\in W(j)}\frac{\omega_{z}\left(f_{i}\right)g_{i,j}}{L_{z}\left({\tilde{f}}_{i}\right)+\varepsilon },\;w_{z,j}=\frac{1}{\left|\partial_{z}f_{j}\right|+\xi },$$

The following equation is solved iteratively:

(27) $$\left(I+\lambda_{2}\Phi_{z}^{k,t}\right)v_{f^{\left(k+2/3\right)}}^{t+1}=v_{f^{\left(k+2/3\right)}},$$

where $v_{f^{\left(k+2/3\right)}}$ is the vectorized form of $f^{\left(k+2/3\right)}$. $\Phi_{z}^{k,t}=C_{z}^{T}U_{z}W_{z}C_{z}$ is the weight matrix of the intermediate result $v_{f^{\left(k+2/3\right)}}^{t+1}$ obtained after *t* iterations of Equation (27) starting from $f^{\left(k+2/3\right)}$, where $C_{z}$ is the Toeplitz matrix of the forward difference operator in the *z* direction, $U_{z}$ and $W_{z}$ are diagonal matrices with diagonal entries $U_{z}\left[j,j\right]=u_{z,j}$ and $W_{z}\left[j,j\right]=w_{z,j}$, and $t=0,1,\ldots ,N_{z}-1$, with $N_{z}$ being the number of iterations for Subproblem 3.

The proposed algorithm is referred to as POCS-PiAwRTV. Its main steps are summarized in Algorithm 1, where $N_{\mathrm{iter}}$ denotes the total number of iterations.
**Algorithm 1:** POCS-PiAwRTV
**Input:** ***p***, $\lambda_{1}$, $\lambda_{2}$, ζ, $N_{\mathrm{iter}}$, $N_{xy}$, $N_{z}$, δ, σ, ε, ξ1    **For**
*k* = 0: $N_{\mathrm{iter}}-1$, **do**2          POCS step represented by Equations (16) and (17)3          **For**
*t* = 0: $N_{xy}-1$, **do**4              Compute the coefficients $u_{x,j}$, $w_{x,j}$, $u_{y,j}$, and $w_{y,j}$ via Equations (20) and (22)5              Solve Equation (24)6          **End for**7          **For**
*t* = 0: $N_{z}-1$, **do**8              Compute the coefficients $u_{z,j}$ and $w_{z,j}$ via Equation (26)9              Solve Equation (27)10        **End for**11  **End for****Output:** the reconstructed image $f^{\left(N_{\mathrm{iter}}\right)}$

## 4. Experiment Results

**Figure 3:**
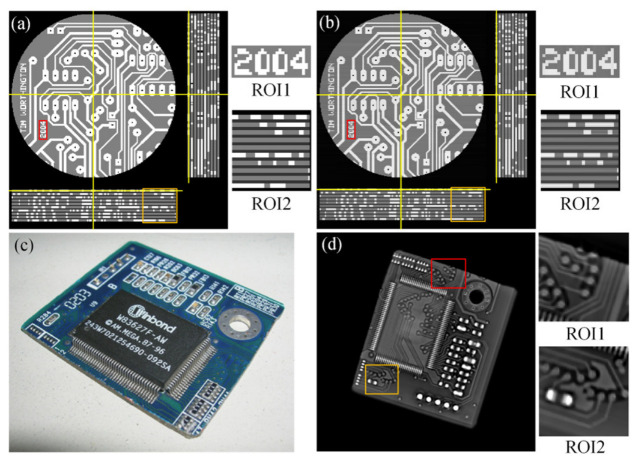
(**a**) PCB phantom. (**b**) Prior image for the simulated data experiment. (**c**) PCB sample. (**d**) Prior image for the real data experiment.

Experiments are performed on a PCB phantom and a real PCB sample. The PCB phantom is constructed to simulate the multi-layer wiring structure of a 10-layer printed circuit board. The PCB phantom is generated by assigning different linear attenuation coefficients to copper traces, insulating layers, and air regions according to the designed wiring pattern, and simulated projection data are obtained using a ray-driven forward projector based on the geometry in Table 1. Figure 3a shows the horizontal, coronal, and sagittal slices of the PCB phantom, where yellow crosshairs indicate the spatial correspondence among the sections. Two regions of interest (ROIs), marked by red and orange bounding boxes, are extracted and magnified to demonstrate local structural details. For the real PCB sample, projection data are acquired using a cone-beam CL system with the parameters listed in Table 1, and all projections are preprocessed with flat-field correction and log-transformation before reconstruction. The real PCB sample is presented in Figure 3c. The proposed POCS-PiAwRTV method is compared with POCS-TV, POCS-RTV, POCS-AAR, PCTV, and PICCS. For the simulated data experiment, root mean square error (RMSE) and gradient magnitude similarity deviation (GMSD) are adopted as quantitative evaluation metrics.

RMSE is defined as:

(28) $$\mathrm{RMSE}=\sqrt{\frac{1}{N}\sum_{i=1}^{N}{\left(x_{i}-y_{i}\right)}^{2}},$$

where *x_i_* and *y_i_* denote the pixel intensities of the reconstructed and ground-truth images, respectively, and *N* is the total number of pixels. A lower RMSE indicates better pixel-wise intensity accuracy.

GMSD [38] is defined as:

(29) $$\mathrm{GMSD}=\sqrt{\frac{1}{N}\sum_{i=1}^{N}{\left(\mathrm{GMS}\left(i\right)-\mathrm{GMSM}\right)}^{2}},$$

where GSM(*i*) is the gradient magnitude similarity at pixel *i*, defined as:

(30) $$\mathrm{GMS}\left(i\right)=\frac{2m_{g}\left(i\right)m_{r}\left(i\right)+C}{m_{g}^{2}\left(i\right)m_{r}^{2}\left(i\right)+C}$$

where *m_g_* and *m_r_* are the gradient magnitude maps of the ground-truth and reconstructed images, respectively, and *C* is a small positive constant to prevent numerical instability. GMSM is the mean of the GMS map over all pixels, defined as:

(31) $$\mathrm{GMSM}=\frac{1}{N}\sum_{i=1}^{N}\mathrm{GMS}\left(i\right).$$

A lower GMSD indicates better preservation of structural and edge information. In this work, GMSD is computed on both the horizontal (xy) and coronal (xz) planes to evaluate edge preservation and inter-slice aliasing suppression, respectively.

The prior image is the POCS-AAR reconstruction with 360 views, as shown in Figure 3b. For the real data experiment, since the ground truth is unavailable, the reconstruction quality is evaluated via visual assessment, and its corresponding prior image is obtained via POCS-AAR reconstruction from 360 projection views, as shown in Figure 3d. All experiments are conducted on a Dell Precision T3680 workstation equipped with an Intel Core i9-14900K processor and an NVIDIA RTX 4090 GPU, with the algorithm implemented in MATLAB R2022b. Among the compared methods, POCS-TV is realized based on the open-source TIGRE toolbox [39].

### 4.1. Simulation Data Experiment

#### 4.1.1. Reconstruction Under Different Sparse Projection Views

**Figure 4:**
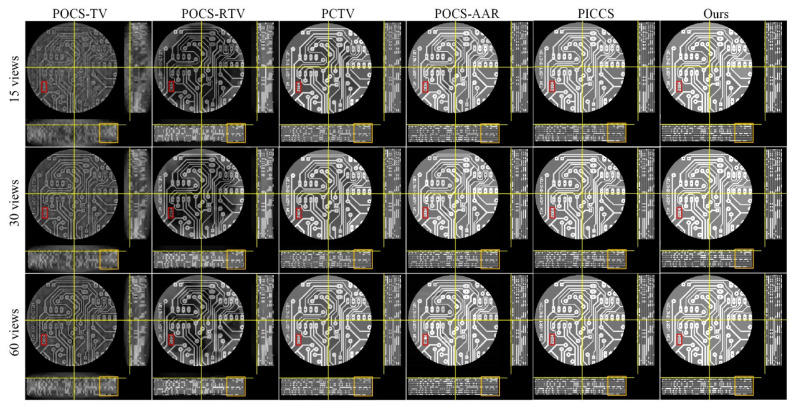
Reconstruction results for PCB phantom under different projection views. The display window is [0, 1].

**Figure 5:**
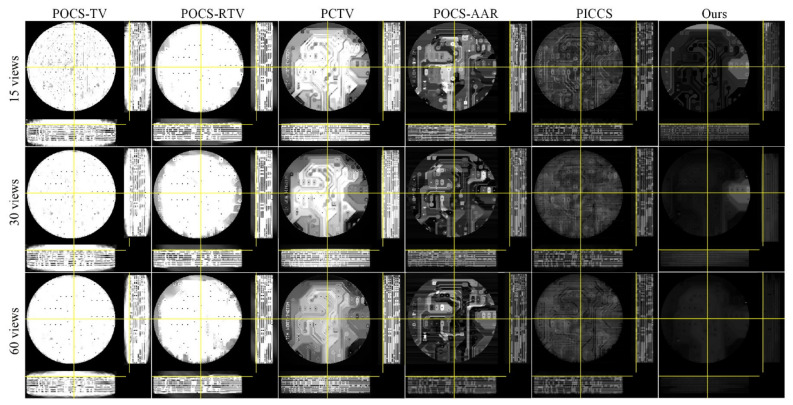
Absolute error maps relative to the ground truth of PCB phantom corresponding to the reconstruction results in Figure 4. The display window is [0, 0.2].

**Figure 6:**
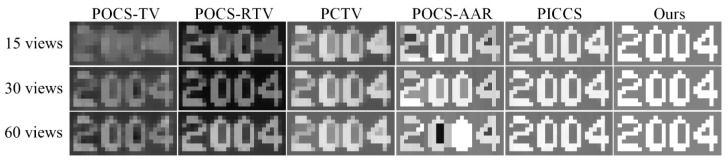
Zoomed-in ROI1 views of the reconstructed PCB phantom. The display window is [0, 1].

**Figure 7:**
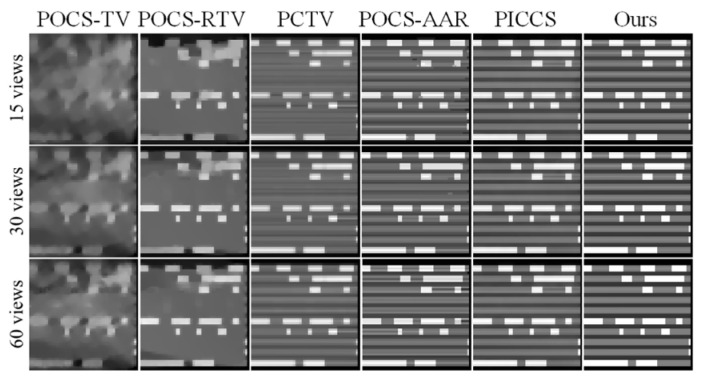
Zoomed-in ROI2 views of the reconstructed PCB phantom. The display window is [0, 1].

**Figure 8:**
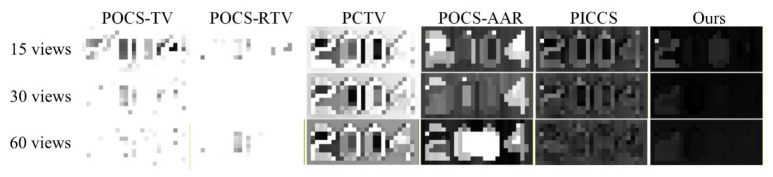
Absolute error maps relative to the ground truth of PCB phantom corresponding to the zoomed-in ROI1 reconstruction results in Figure 6. The display window is [0, 0.2].

**Figure 9:**
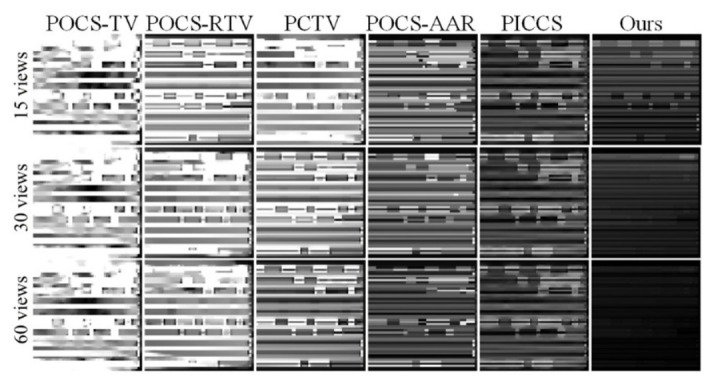
Absolute error maps relative to the ground truth of PCB phantom corresponding to the zoomed-in ROI2 reconstruction results in Figure 7. The display window is [0, 0.2].

**Figure 10:**
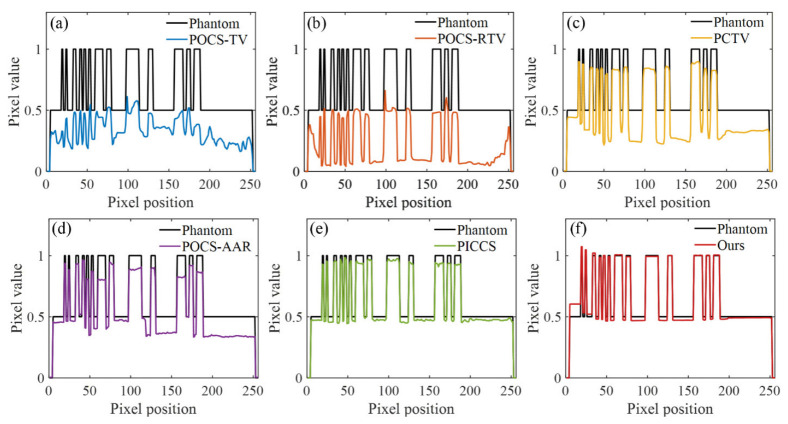
Horizontal profiles of the 128th column for the reconstructed PCB phantom under 15 projection views. (**a**) POCS-TV; (**b**) POCS-RTV; (**c**) PCTV; (**d**) POCS-AAR; (**e**) PICCS; (**f**) Ours.

**Figure 11:**
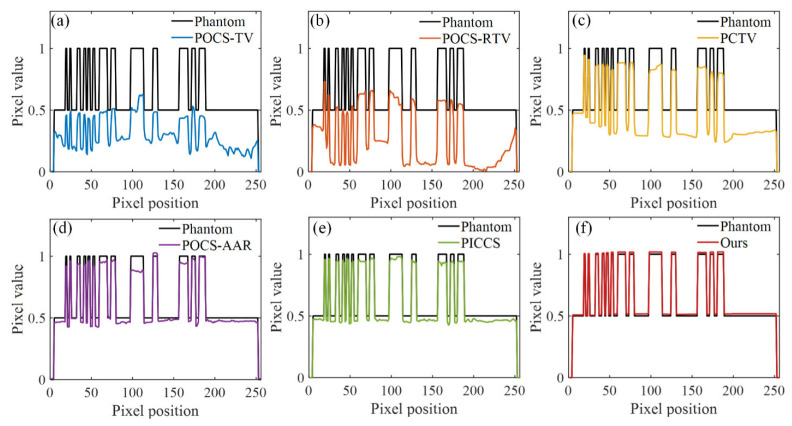
Horizontal profiles of the 128th column for the reconstructed PCB phantom under 30 projection views. (**a**) POCS-TV; (**b**) POCS-RTV; (**c**) PCTV; (**d**) POCS-AAR; (**e**) PICCS; (**f**) Ours.

**Figure 12:**
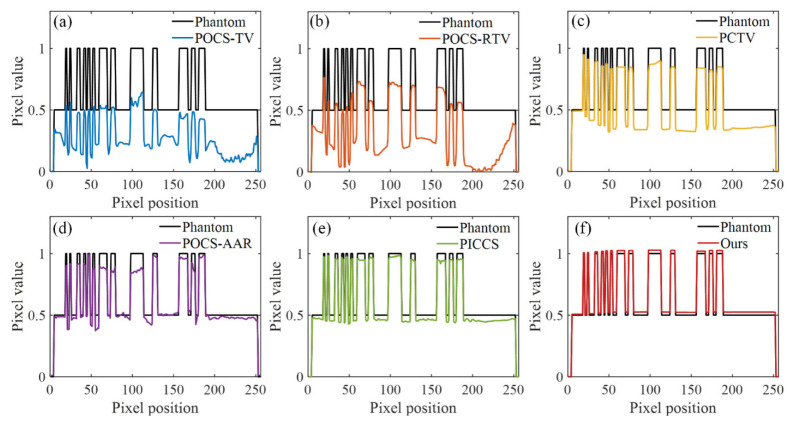
Horizontal profiles of the 128th column for the reconstructed PCB phantom under 60 projection views. (**a**) POCS-TV; (**b**) POCS-RTV; (**c**) PCTV; (**d**) POCS-AAR; (**e**) PICCS; (**f**) Ours.

**Figure 13:**
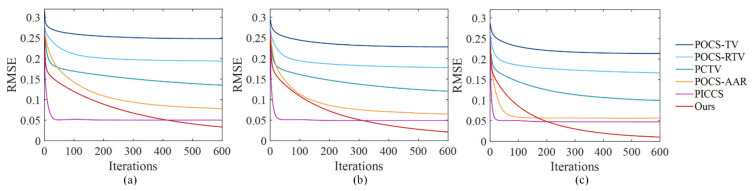
RMSE of the reconstruction results for PCB phantom at each iteration. (**a**) 15 views; (**b**) 30 views; (**c**) 60 views.

Experiments are performed on a PCB phantom with 15, 30, and 60 projection views. To eliminate subjective parameter bias and ensure fair comparison, this study adopts a systematic parameter tuning strategy from the literature [40]. Firstly, preliminary tests are conducted to define reasonable value intervals for key tunable hyperparameters, including $\lambda_{1}$, $\lambda_{2}$, δ, and σ. Subsequently, a grid search algorithm is implemented to search for the optimal parameter combination, where candidate groups are assessed comprehensively via multiple quantitative evaluation metrics. Other secondary auxiliary parameters, such as ε, ξ, $N_{xy}$, and $N_{z}$, are set to fixed values according to conventional practices. In the simulation data experiment, the parameters of the proposed algorithm are set as follows: $\lambda_{1}=0.05$, $\lambda_{2}=0.005$ for 15 projections; $\lambda_{1}=0.02$, $\lambda_{2}=0.002$ for 30 projections; and $\lambda_{1}=0.01$, $\lambda_{2}=0.001$ for 60 projections, while the remaining parameters are uniformly set to ζ=0.99, $N_{xy}=3$, $N_{z}=3$, σ=3, δ=0.1, ε=0.001, and ξ=0.01. The comparison methods are optimized within reasonable ranges, and all methods perform 600 iterations, at which point the RMSE curves stabilize. Figure 4 illustrates the reconstruction results of all algorithms under different numbers of projection views. As the number of projections increases, the reconstruction quality of all methods is progressively improved. POCS-TV produces blurry reconstructions with over-smoothed wiring edges and loss of details. Aliasing artifacts are evident in cross-sectional slices, and fine structures cannot be fully recovered even with 60 projection views. POCS-RTV restores wiring contours with clearer edges. Nevertheless, it suffers from uneven gray patches and inter-slice blurring. PCTV reconstructs clear wiring edges and distinct layered structures. However, gaps between adjacent wiring layers are not recovered, and the horizontal slice still exhibits detail blurring and local grayscale distortion. POCS-AAR provides better contrast and higher structural integrity, and inter-slice aliasing is effectively suppressed. However, some circuit details remain unrecovered, and a few streak artifacts persist in the horizontal slice. PICCS delivers high-resolution reconstructions characterized by sharp circuit edges and well-preserved structures, but the overall grayscale level is low, resulting in a dark appearance. The proposed algorithm achieves competitive reconstruction quality across all projection views. Circuit structures, fine details, and board text are clearly recovered, and the algorithm performs well even under extremely sparse sampling conditions. Figure 5 presents the corresponding absolute error maps relative to the ground truth, where the error maps clearly show that our method yields the smallest residual errors across all projection views. To further verify the reconstruction performance of each algorithm on local features, two ROIs are selected and enlarged, as shown in Figure 6 and Figure 7. In Figure 6, POCS-TV and POCS-RTV yield reconstructions with blurred text edges and heavily merged strokes. Although PCTV, POCS-AAR, and PICCS reconstruct distinguishable text contours, they still suffer from blurred stroke details and insufficient contrast. In Figure 7, the reconstruction results of POCS-TV and POCS-RTV show severe structural blurring. PCTV reconstructs clear layered wiring structures but fails to recover interlayer gaps. POCS-AAR and PICCS achieve limited improvement with blurring of delicate structures. The proposed algorithm yields desirable reconstruction performance in both ROIs. Printed text strokes are clear, and interlayer gaps along with fine structures are well recovered, outperforming all other algorithms in local detail restoration. The corresponding absolute error maps in Figure 8 and Figure 9 further confirm these observations, showing that our method produces the lowest residual errors in the ROI regions, particularly at structural edges and fine details. Figure 10, Figure 11 and Figure 12 present the horizontal profiles of the 128th column for the reconstructed images under 15, 30, and 60 projection views, respectively. In all cases, the profiles of the proposed algorithm are highly consistent with the ground truth, demonstrating accurate grayscale intensity recovery. Table 2 lists the quantitative metrics, where the proposed algorithm achieves favorable results across all evaluation metrics. Paired *t*-tests confirm that the improvements of the proposed method over all competing methods in Table 2 are statistically significant (*p* < 0.05). Figure 13 presents the RMSE convergence curves, showing that, when all methods reach convergence, the proposed algorithm attains the minimum RMSE.

#### 4.1.2. Robustness to Projection Noise

**Figure 14:**
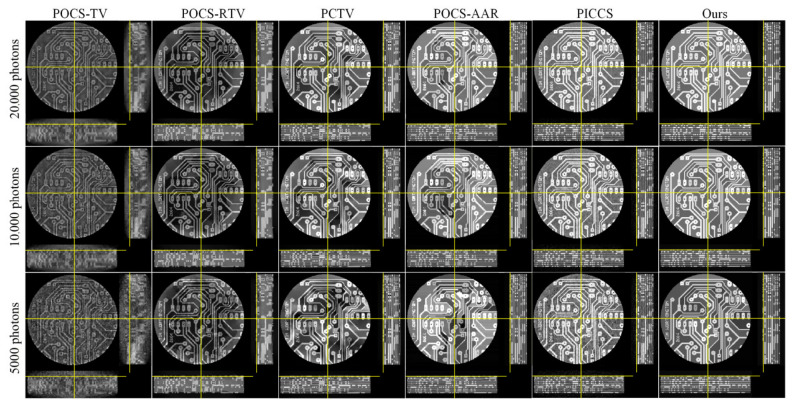
Reconstruction results for PCB phantom under different Poisson noise levels in projection data. The display window is [0, 1].

To evaluate the robustness of the proposed method against noise in projection data, we perform experiments under 30 projection views with three different photon counts of 5000, 10,000, and 20,000. Lower photon counts correspond to higher Poisson noise levels. The regularization parameters are adjusted according to the noise level: for 20,000 photons, $\lambda_{1}=0.03$, $\lambda_{2}=0.003$; for 10,000 photons, $\lambda_{1}=0.05$, $\lambda_{2}=0.005$; and for 5000 photons, $\lambda_{1}=0.08$ and $\lambda_{2}=0.008$. Figure 14 shows the reconstruction results under different photon counts for all competing methods and the proposed method. As the photon count decreases, the noise level in the reconstructed images increases for all methods. POCS-TV and POCS-RTV produce severe noise and blurring under low photon counts, failing to recover fine structures. PCTV and POCS-AAR show moderate improvement but still suffer from visible artifacts. PICCS effectively recovers the main structures without noticeable artifacts, but residual noise remains visible, especially under low photon counts. In contrast, the proposed method consistently produces cleaner reconstructions with better edge and detail preservation across all noise levels. Table 3 reports the quantitative metrics. The proposed method consistently outperforms all competing methods across all photon counts. This quantitatively confirms that the proposed method is highly robust to Poisson noise in projection data.

#### 4.1.3. Robustness to Prior Image Degradation

**Figure 15:**
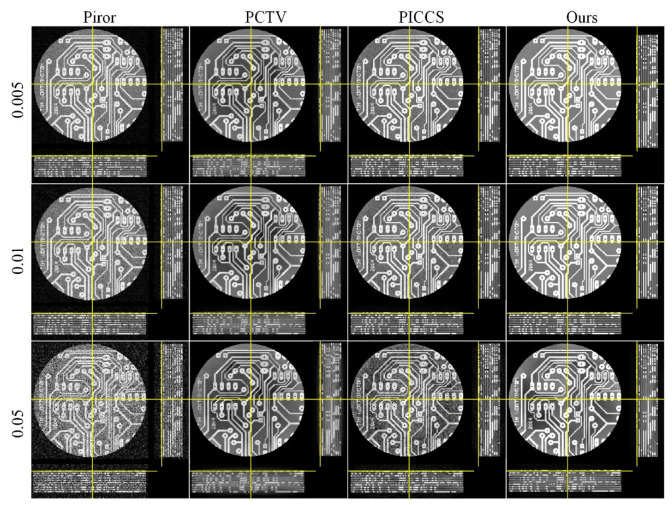
Reconstruction results for PCB phantom under different Gaussian noise variances in prior image. The leftmost column shows the prior images with added noise. The display window is [0, 1].

To assess the impact of prior image quality on reconstruction performance, we add Gaussian noise with variances of 0.005, 0.01, and 0.05 to the prior image under 30 projection views. Higher variances indicate more severe degradation. The regularization parameters are adjusted according to the noise variance: for 0.005, $\lambda_{1}=0.025$, $\lambda_{2}=0.0025$; for 0.01, $\lambda_{1}=0.03$, $\lambda_{2}=0.003$; and for 0.05, $\lambda_{1}=0.035$, $\lambda_{2}=0.0035$. Figure 15 displays the degraded prior images (leftmost column) and the corresponding reconstructions from PCTV, PICCS, and the proposed method. As the noise variance increases, the quality of the prior image deteriorates, leading to performance degradation for all methods. PCTV produces blurry reconstructions with noticeable artifacts under all noise levels. PICCS achieves better results but still suffers from structural degradation as the prior image quality worsens. Our method consistently delivers better structure preservation and fewer artifacts than PCTV and PICCS, confirming its resilience to prior image degradation. Table 4 lists the quantitative metrics. The proposed framework achieves superior metric scores to PCTV and PICCS for every noise variance configuration, quantitatively demonstrating its outstanding stability when facing low-quality prior images.

#### 4.1.4. Detection of Local Defects with Defect-Free Prior

**Figure 16:**
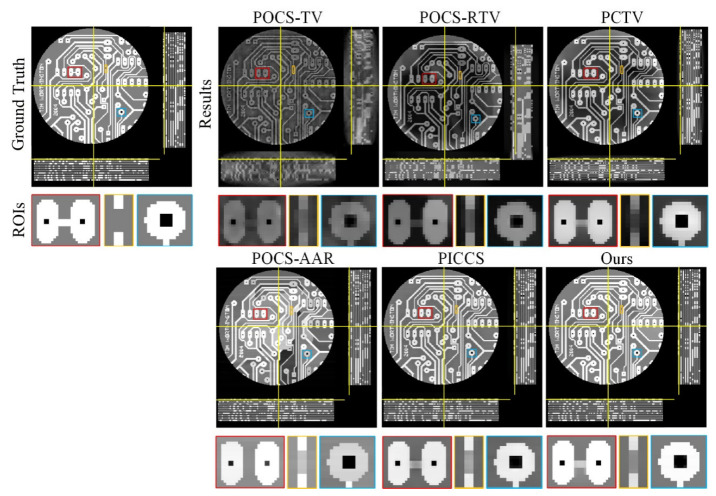
Reconstruction results of the defective PCB phantom using a defect-free prior image. The leftmost column shows the ground truth of the defective phantom. The display window is [0, 1].

**Figure 17:**
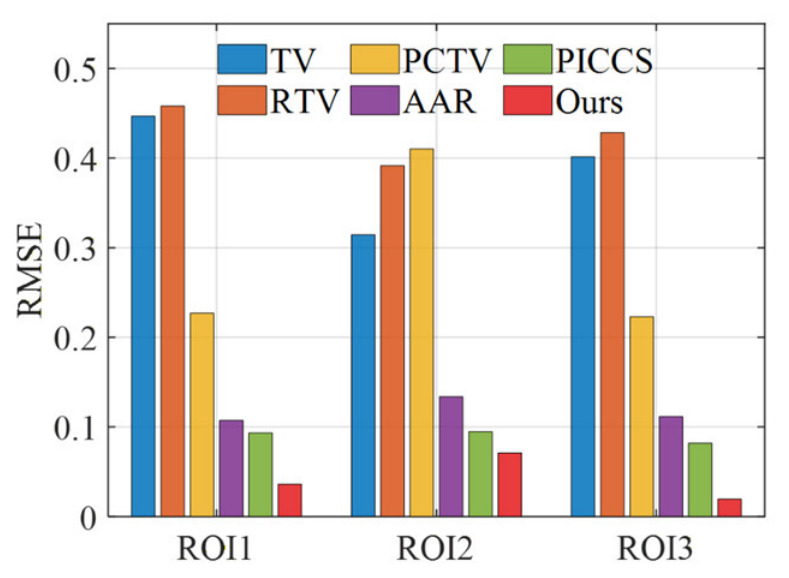
ROI-wise RMSE of different algorithms for the defective PCB phantom with a defect-free prior.

To evaluate whether the proposed method erroneously eliminates local defects when a defect-free prior image is used, we perform experiments on a defective PCB phantom under 30 projection views. Three types of representative defects are introduced into the phantom: short circuits, open circuits, and via size anomalies. The prior image is reconstructed from a defect-free phantom. Figure 16 shows the ground truth (leftmost column) and the reconstruction results of all competing methods, along with three zoomed-in ROIs for detailed visual comparison. POCS-TV and POCS-RTV produce blurry reconstructions that fail to preserve the defect structures. PCTV and POCS-AAR show moderate improvement but still suffer from noticeable structural distortion in the defect regions. PICCS achieves relatively better defect preservation, yet some fine details remain degraded. In contrast, the proposed method successfully preserves the local defects with clear edges and fine structural details, demonstrating that the defect-free prior does not cause the algorithm to smooth out anomalies. Table 5 reports the quantitative metrics. The proposed method achieves the best performance across all metrics. Figure 17 shows the ROI-wise RMSE. Our method achieves the lowest RMSE values across all three metrics and all ROIs, further validating its robustness in local defect preservation.

### 4.2. Real Data Experiment

**Figure 18:**
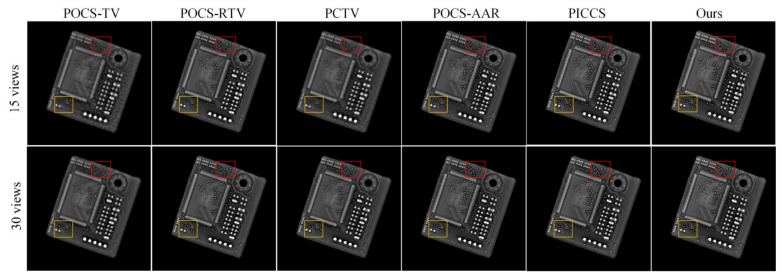
Reconstruction results of real PCB sample obtained by different algorithms. The display window is [0, 0.25].

**Figure 19:**
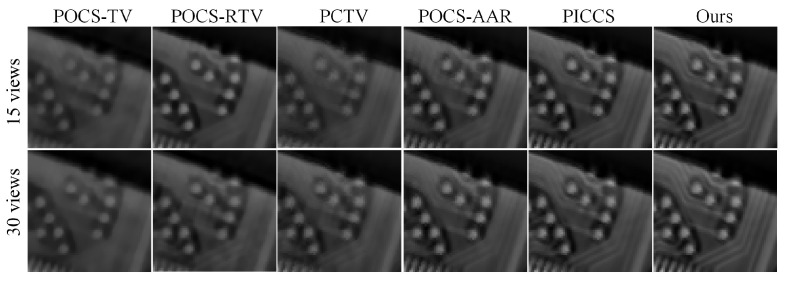
Zoomed-in ROI1 views of the reconstructed PCB sample. The display window is [0, 0.25].

**Figure 20:**
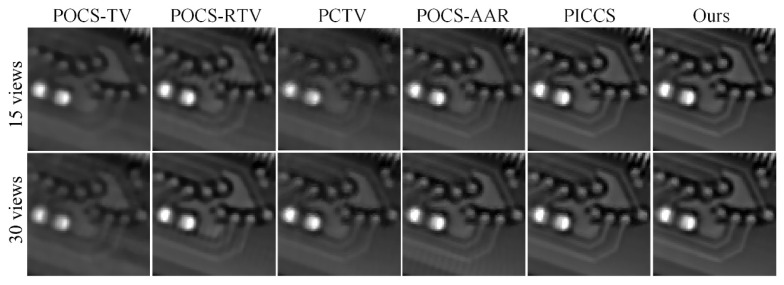
Zoomed-in ROI2 views of the reconstructed PCB sample. The display window is [0, 0.25].

**Figure 21:**
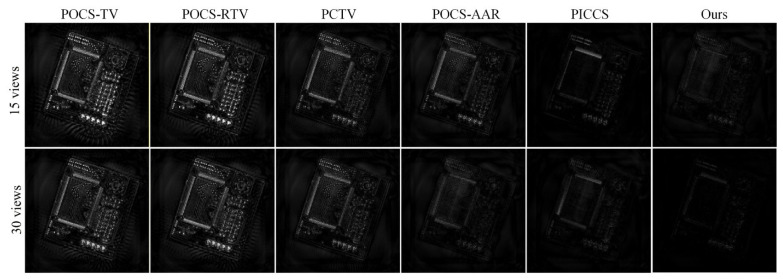
Absolute error maps relative to the prior image corresponding to the reconstruction results in Figure 18. The display window is [0, 0.1].

**Figure 22:**
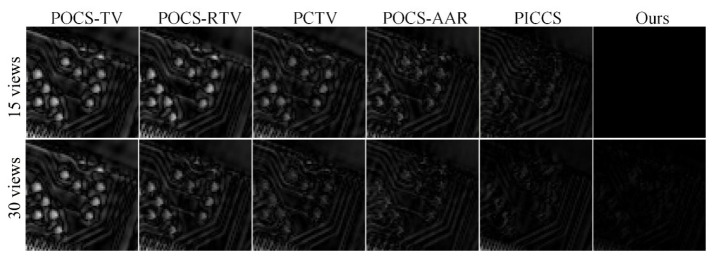
Absolute error maps relative to the prior image corresponding to the zoomed-in ROI1 reconstruction results in Figure 19. The display window is [0, 0.1].

**Figure 23:**
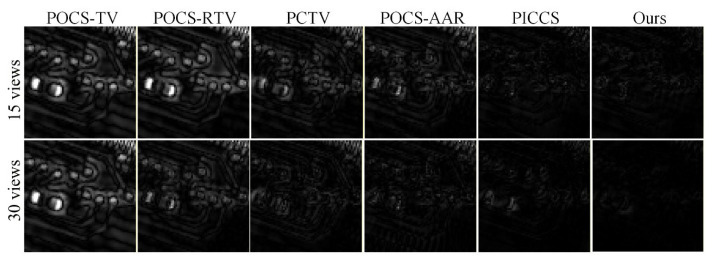
Absolute error maps relative to the prior image corresponding to the zoomed-in ROI2 reconstruction results in Figure 20. The display window is [0, 0.1].

Experiments are performed on a real PCB sample with 15 and 30 views. The parameters for the real data experiment are set as follows: $\lambda_{1}=0.00001$, $\lambda_{2}=0.000001$ for 15 projections; $\lambda_{1}=0.000025$, $\lambda_{2}=0.0025$ for 30 projections, while the remaining parameters are uniformly set to ζ=0.99, $N_{xy}=3$, $N_{z}=3$, σ=3, δ=0.1, ε=0.001, and ξ=0.01. For the other algorithms, the parameters are optimally tuned within reasonable ranges to ensure a fair comparison. The total number of iterations for each iterative algorithm is set to 50, as the relative change between consecutive iterations falls below 10^−4^ and further iterations yield negligible improvement. Figure 18, Figure 19 and Figure 20 present the 45th horizontal slice of the reconstructions obtained by different methods, as well as the magnified views of two ROIs in this slice. To further evaluate the deviation of each method from the prior image, Figure 21, Figure 22 and Figure 23 present the corresponding absolute error maps between the reconstructions and the prior image for the full slice, ROI 1, and ROI 2, respectively. The reconstruction quality of all algorithms improves as the number of projection views increases. Comprehensive analysis of both global and local results demonstrates that POCS-TV, POCS-RTV, and PCTV yield blurry reconstructions with over-smoothed wiring edges and severe detail loss. Furthermore, adjacent wiring traces exhibit noticeable adhesion. POCS-AAR is capable of roughly identifying the main contours of the wiring structures. However, obvious structural blurring remains, and fine interlayer gaps between adjacent structures cannot be effectively recovered. PICCS yields reconstructions with clearer wiring structures. Nevertheless, the overall low grayscale intensity and insufficient contrast impair the visibility of delicate structures, which still requires further improvement. The proposed algorithm achieves favorable reconstruction quality under both 15 and 30 projection views, with well-preserved global structures and uniform grayscale distribution. In both ROIs, the dense wiring boundaries are clear with no adhesion. Solder joint structures and fine gaps among adjacent wiring traces are accurately recovered. The proposed method surpasses all competing algorithms in local detail restoration. The absolute error maps in Figure 21, Figure 22 and Figure 23 further confirm these observations. Our method consistently shows the smallest deviations from the prior image, especially at edges and fine features.

## 5. Discussions

### 5.1. Parameter Setting and Sensitivity Analysis

Reasonable parameter selection is crucial for obtaining high-quality reconstruction results. There are substantial discrepancies between simulated and real data in noise characteristics, voxel ranges, and structural complexity, necessitating distinct optimal parameters. Owing to intricate structures and smaller intensity ranges in real data, smaller regularization factors are typically required to avoid over-smoothing. The parameter ranges are set to $\lambda_{1},\lambda_{2}\in \left[0.0001,0.1\right]$ for simulation experiments and $\lambda_{1},\lambda_{2}\in \left[1\times 10^{-7},1\times 10^{-4}\right]$ for real data. Though grid search is computationally costly, one calibration suffices for all samples of the same type. $\lambda_{1}$ controls the strength of the 2D PiAwRTV in the horizontal direction to maintain in-plane structures, while $\lambda_{2}$ adjusts the strength of 1D PiAwRTV constraint in the vertical direction for suppressing inter-slice aliasing and cone-beam artifacts. Insufficient values of these two parameters lead to inadequate detail preservation and blurred edges, whereas excessively large values tend to introduce blocky artifacts. In this study, the parameter selection principle of $\lambda_{1}>\lambda_{2}$ is adopted. Stronger regularization is imposed along the horizontal direction with reliable gradient information to enhance structure retention, while milder constraints are applied in the vertical direction to avoid erroneous reconstruction updates caused by artifacts. Experimental verification shows that setting $\lambda_{2}$ to approximately 0.1 times $\lambda_{1}$ is appropriate. Furthermore, when additional noise is added to either the projection data or the prior image, the regularization factors should be moderately increased to effectively suppress noise-induced artifacts. The PiAwRTV regularization term involves the parameters σ, δ, ε, ξ, $N_{xy}$ and $N_{z}$. δ is used to adjust the sensitivity of the adaptive weights, enabling the algorithm to impose weaker penalties on strong edges and stronger penalties in flat regions. Experimental results show that δ yields favorable reconstruction performance within the range of 0.05–2. In this study, δ is set to 0.1 to balance stability and generalizability. σ represents the standard deviation of the Gaussian kernel, which dominates the perception scope of local structures. Its value is associated with image resolution and target structure scale. Considering both simulated and real PCB structural characteristics, σ is set to 3, ensuring stable local feature capture. ε and ξ are small constants to prevent division by zero. Their variations within 0.0001–0.01 exert negligible influence on reconstruction quality. In this study, ε is set to 0.001, and ξ is set to 0.01. $N_{xy}$ and $N_{z}$ are the iteration numbers for solving Subproblem 2 and Subproblem 3, respectively. Setting both to 3 provides a trade-off between subproblem convergence and algorithmic efficiency. $N_{\mathrm{iter}}$ is the total number of algorithm iterations, and its value should be chosen to allow the algorithm to reach a steady state.

**Figure 24:**
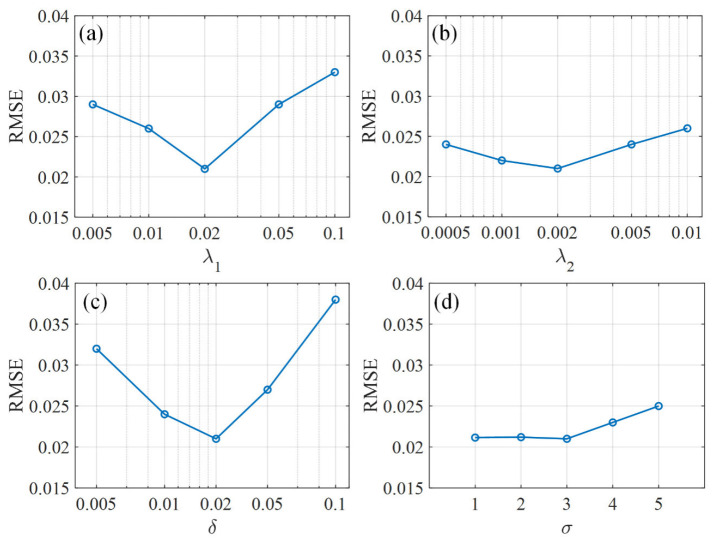
Parameter sensitivity analysis under 30 views. (**a**–**d**) RMSE with respect to $\lambda_{1}$, $\lambda_{2}$, δ, and σ.

A sensitivity analysis of parameters $\lambda_{1}$, $\lambda_{2}$, δ, and σ is conducted under 30 projection views, where each parameter is varied individually within a reasonable range around its optimal value while the others are kept fixed. As shown in Figure 24, RMSE remains stable under these variations, confirming that the performance improvements are not due to overfitting to specific parameter values.

### 5.2. Computational Time

Table 6 reports the computational time of all algorithms under 30 projection views. All methods share the same GPU-accelerated projection and backprojection operations via the TIGRE toolbox. POCS-TV benefits from full GPU acceleration for both projection/backprojection and TV regularization. In contrast, POCS-RTV, PCTV, POCS-AAR, PICCS, and the proposed method compute their regularization terms on the CPU. POCS-TV achieves the shortest total time due to its simple TV regularization and full GPU acceleration. POCS-RTV and PCTV require more complex regularization on the CPU, resulting in moderately longer computation times. POCS-AAR and PICCS involve iterative optimization with additional constraints, leading to higher computational costs. The proposed method is slightly slower than POCS-AAR, primarily because the 2D in-plane regularization and adaptive weighting are more computationally intensive than the 1D regularizations used in AAR. A detailed per-iteration analysis shows that the regularization accounts for approximately 70% of the total computation time in our method.

Regarding memory usage, the GPU memory consumption of the proposed method is approximately 0.3 GB for a 256 × 75 × 256 volume under 30 projections, as the regularization terms are computed on the CPU. The per-iteration computational complexity is approximately O(*N*), where *N* is the number of voxels, as the regularization operations are performed voxel-wise. The total time scales approximately linearly with the volume size. In our experiments, the real data volume is approximately five times larger than the simulation volume, and the per-iteration time increases by a factor of about 3.3, which is reasonably close to linear scaling. The sub-linear scaling is mainly due to the improved utilization of GPU cores for larger volumes and the fixed overhead that does not increase with volume size.

For industrial inspection, the current total time of approximately 30 min for real data is acceptable for offline quality control. Moreover, although the proposed method is slightly slower than some competing methods, its superior artifact suppression and edge preservation capabilities justify the longer runtime. The regularization terms are highly parallelizable, and porting them to GPU is expected to reduce the total time by a factor of 5–10, making it competitive with or faster than POCS-AAR and PICCS.

### 5.3. Ablation Experiments

To quantitatively evaluate the contribution of each core component in the proposed PiAwRTV model, we conduct ablation experiments on the PCB phantom under 30 projection views. Three variants are evaluated:

(1) Fixed Weights, where the adaptive weights $\omega_{x}\left(f_{i}\right)$, $\omega_{y}\left(f_{i}\right)$, and $\omega_{z}\left(f_{i}\right)$ are replaced with fixed weights ω = 1;
(2) No Prior, where the prior image is not used and the WIV is computed from the current image instead of the prior image;
(3) 3D Regularization, where the regularization is applied uniformly in 3D without 2D/1D decoupling.

The complete model is denoted as Full Model.

**Figure 25:**
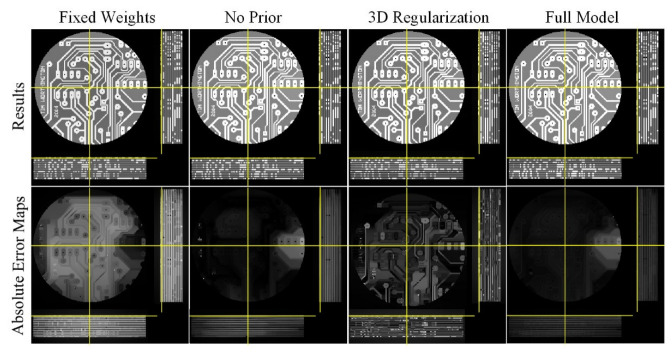
Reconstruction results (display window [0, 1]) and absolute error maps (display window [0, 0.2]) relative to the ground truth of the PCB phantom.

Figure 25 and Table 7 present the ablation study results. The full model produces the clearest structures with the fewest artifacts. Removing adaptive weights causes the most severe degradation, with the most significant discrepancies in the error map and the poorest quantitative performance. Replacing anisotropic decomposition with isotropic regularization primarily affects through-plane quality, as reflected by the substantial increase in GMSD_xz_. Removing the prior image leads to a moderate degradation, confirming the contribution of prior information, while the method remains effective without it.

## 6. Conclusions

To address the problems of aliasing artifacts and structural blurring in sparse-view CL, this paper proposes the PiAwRTV reconstruction model. Built upon the conventional RTV model, the proposed method incorporates high-quality prior images as global structural prior information and constructs local gradient-driven adaptive weights to dynamically modulate anisotropic regularization strength. Meanwhile, the regularization constraint is decoupled into two-dimensional horizontal and one-dimensional vertical components. The algorithm adopts an alternating minimization strategy that decomposes the optimization problem into three subproblems. Experimental results on both simulated and real datasets verify that the proposed method maintains clear in-plane structures while effectively suppressing inter-slice aliasing artifacts. The proposed method is particularly suitable for industrial nondestructive testing scenarios where a high-quality prior image of a standard defect-free sample is available, for example, obtained through prior scanning or CAD design, which is common in batch inspection in manufacturing and consistent with the typical application scenario of prior image-guided reconstruction methods. While the current validation focuses on PCB structures, the method is general and expected to apply to other plate-like objects. Future work will extend the validation to a broader range of materials and applications, including composite materials and biomedical structures. Although the proposed algorithm achieves superior reconstruction performance compared with existing methods, it still possesses certain limitations. As demonstrated in the prior image degradation experiments in Section 4.1.4, the method remains robust under moderate prior image degradation, but its performance may decline under severe prior image errors. To further enhance the robustness and generalization capability of the algorithm, future research will explore the integration of physical models with learning-based strategies, as suggested in [41]. While this direction is promising, the current adoption of such methods is constrained by the limited availability of training data. Our future work will prioritize data collection and dataset construction to support the development of hybrid physical–AI models. Future work will also extend the evaluation to more challenging conditions, including geometric misalignments and severe scattering. Computational efficiency will be further improved through parallel acceleration and fast iterative algorithms.

## Figures and Tables

**Table 1:** Reconstruction and geometric parameters.

Parameters	PCB Phantom	PCB Sample
Reconstruction image size	256 × 256 × 75	500 × 500 × 100
Voxel size (mm^3^)	0.5 × 0.5 × 0.5	0.2 × 0.2 × 0.2
Source-to-object distance (mm)	217	620
Source-to-detector distance (mm)	815	820
Detector size	600 × 600	980 × 980
Detector pixel size (mm^2^)	1 × 1	0.127 × 0.127
Tilt angle φ (°)	45	41

**Table 2:** Quantitative metrics under different projection views by different algorithms.

Projection Views	Algorithm	RMSE ↓ *	GMSD_xy_(×10^−5^) ↓ *	GMSD_xz_(×10^−5^) ↓ *
15	POCS-TV	0.248	55.422	13.493
	POCS-RTV	0.194	31.802	7.0240
	PCTV	0.135	10.901	3.453
	POCS-AAR	0.078	3.837	1.723
	PICCS	0.051	1.922	0.797
	Ours	0.034	0.605	0.296
30	POCS-TV	0.228	48.257	9.374
	POCS-RTV	0.178	25.322	5.491
	PCTV	0.120	7.894	2.068
	POCS-AAR	0.065	2.880	1.106
	PICCS	0.049	1.694	0.617
	Ours	0.021	0.240	0.051
60	POCS-TV	0.213	43.248	8.188
	POCS-RTV	0.166	21.604	4.541
	PCTV	0.099	6.361	1.865
	POCS-AAR	0.057	1.942	1.492
	PICCS	0.047	1.503	0.533
	Ours	0.010	0.053	0.013

* ↓ indicates that lower values represent better performance.

**Table 3:** Quantitative metrics under different Poisson noise levels in projection data by different algorithms.

Photon Count	Algorithm	RMSE ↓ *	GMSD_xy_(×10^−5^) ↓ *	GMSD_xz_(×10^−5^) ↓ *
20,000	POCS-TV	0.230	48.617	9.738
	POCS-RTV	0.185	28.107	6.208
	PCTV	0.130	9.356	1.680
	POCS-AAR	0.072	3.432	2.634
	PICCS	0.058	3.347	1.044
	Ours	0.049	2.230	0.438
10,000	POCS-TV	0.233	49.030	10.443
	POCS-RTV	0.187	28.584	6.496
	PCTV	0.139	10.970	2.881
	POCS-AAR	0.090	4.566	4.352
	PICCS	0.069	5.252	1.711
	Ours	0.063	3.050	0.809
5000	POCS-TV	0.241	49.461	11.774
	POCS-RTV	0.192	28.920	7.279
	PCTV	0.155	13.175	5.327
	POCS-AAR	0.113	10.173	7.495
	PICCS	0.090	7.615	2.998
	Ours	0.081	5.539	1.377

* ↓ indicates that lower values represent better performance.

**Table 4:** Quantitative metrics under different Gaussian noise variances in prior image by different algorithms.

Noise Variance	Algorithm	RMSE ↓ *	GMSD_xy_(×10^−5^) ↓ *	GMSD_xz_(×10^−5^) ↓ *
0.005	PCTV	0.124	11.491	2.999
	PICCS	0.058	2.360	0.863
	Ours	0.023	0.329	0.073
0.01	PCTV	0.13	13.072	3.527
	PICCS	0.067	3.076	1.205
	Ours	0.026	0.491	0.193
0.05	PCTV	0.155	19.949	3.926
	PICCS	0.105	7.590	3.320
	Ours	0.080	5.074	2.633

* ↓ indicates that lower values represent better performance.

**Table 5:** Quantitative metrics of the reconstruction results for the defective PCB phantom using a defect-free prior image by different algorithms.

Algorithm	RMSE ↓ *	GMSD_xy_(×10^−5^) ↓ *	GMSD_xz_(×10^−5^) ↓ *
POCS-TV	0.231	49.257	9.574
POCS-RTV	0.181	25.322	5.491
PCTV	0.128	7.894	2.068
POCS-AAR	0.071	2.880	1.107
PICCS	0.050	1.695	0.617
Ours	0.036	0.240	0.051

* ↓ indicates that lower values represent better performance.

**Table 6:** The computational time of different algorithms.

Data	Algorithm	Runtime per Iteration (s)	Iteration Number	Total Runtime (min)
PCB phantom	POCS-TV	4.61	600	46.1
	POCS-RTV	8.56	600	85.6
	PCTV	6.83	600	68.3
	POCS-AAR	10.52	600	105.2
	PICCS	9.54	600	95.4
	Ours	11.00	600	110.0
PCB sample	POCS-TV	15.20	50	12.7
	POCS-RTV	26.80	50	22.3
	PCTV	22.10	50	18.4
	POCS-AAR	31.50	50	26.3
	PICCS	29.80	50	24.8
	Ours	36.00	50	30.0

**Table 7:** Quantitative comparison of ablation study results under 30 projection views.

Algorithm	RMSE ↓ *	GMSD_xy_(×10^−5^) ↓ *	GMSD_xz_(×10^−5^) ↓ *
Fixed Weights	0.071	2.599	0.549
No Prior	0.033	0.573	0.108
3D Regularization	0.051	2.007	2.048
Full Model	0.021	0.240	0.051

* ↓ indicates that lower values represent better performance.

## Data Availability

Data are available on request from the authors.
